# Increased red cell distribution width in Fanconi anemia: a novel marker of stress erythropoiesis

**DOI:** 10.1186/s13023-016-0485-0

**Published:** 2016-07-25

**Authors:** Rosa Sousa, Cristina Gonçalves, Isabel Couto Guerra, Emília Costa, Ana Fernandes, Maria do Bom Sucesso, Joana Azevedo, Alfredo Rodriguez, Rocio Rius, Carlos Seabra, Fátima Ferreira, Letícia Ribeiro, Anabela Ferrão, Sérgio Castedo, Esmeralda Cleto, Jorge Coutinho, Félix Carvalho, José Barbot, Beatriz Porto

**Affiliations:** 1 Laboratory of Cytogenetics, Abel Salazar Institute for Biomedical Sciences, University of Porto (ICBAS, UP), Porto, Portugal; 2 Clinical Hematology Service, Hospital Center of Porto (CHP), Porto, Portugal; 3 Pediatric Hematology Unity, Hospital Center of Porto (CHP), Porto, Portugal; 4 Pediatric Hematology-Oncology Unity, Hospital Center of S. João, Porto (CHSJ), Porto, Portugal; 5 Hematology Service, Hospital and University Center of Coimbra (CHUC), Porto, Portugal; 6 Laboratory of Cytogenetics, National Institute of Pediatrics, Ciudad de Mexico (INP), Mexico City, Mexico; 7 Clinical Pathology Service, Infante D. Pedro Hospital, Aveiro (CHBV), Aveiro, Portugal; 8 Hematology Service, Hospital Center of S. João, Porto (CHSJ), Porto, Portugal; 9 Pediatric Service, Hospital Center of Lisboa Norte (CHLN), Lisbon, Portugal; 10 Medical Genetics and Prenatal Diagnosis Prof Doctor Sérgio Castedo, Porto (GDPN), Porto, Portugal; 11 UCIBIO-REQUIMTE, Laboratory of Toxicology, Department of Biological Sciences, Faculty of Pharmacy, University of Porto, Porto, Portugal

**Keywords:** Fanconi anemia (FA), Red cell distribution width (RDW), Stress erythropoiesis, Bone marrow failure (BMF), Oxidative stress (OS)

## Abstract

**Background:**

Red cell distribution width (RDW), a classical parameter used in the differential diagnosis of anemia, has recently been recognized as a marker of chronic inflammation and high levels of oxidative stress (OS). Fanconi anemia (FA) is a genetic disorder associated to redox imbalance and dysfunctional response to OS. Clinically, it is characterized by progressive bone marrow failure, which remains the primary cause of morbidity and mortality. Macrocytosis and increased fetal hemoglobin, two indicators of bone marrow stress erythropoiesis, are generally the first hematological manifestations to appear in FA. However, the significance of RDW and its possible relation to stress erythropoiesis have never been explored in FA. In the present study we analyzed routine complete blood counts from 34 FA patients and evaluated RDW, correlating with the hematological parameters most consistently associated with the FA phenotype.

**Results:**

We showed, for the first time, that RDW is significantly increased in FA. We also showed that increased RDW is correlated with thrombocytopenia, neutropenia and, most importantly, highly correlated with anemia. Analyzing sequential hemograms from 3 FA patients with different clinical outcomes, during 10 years follow-up, we confirmed a consistent association between increased RDW and decreased hemoglobin, which supports the postulated importance of RDW in the evaluation of hematological disease progression.

**Conclusions:**

This study shows, for the first time, that RDW is significantly increased in FA, and this increment is correlated with neutropenia, thrombocytopenia, and highly correlated with anemia. According to the present results, it is suggested that increased RDW can be a novel marker of stress erythropoiesis in FA.

**Electronic supplementary material:**

The online version of this article (doi:10.1186/s13023-016-0485-0) contains supplementary material, which is available to authorized users.

## Background

Red cell distribution width (RDW) is a simple hematological parameter routinely obtained in standard complete blood cell counts, being currently used in the differential diagnosis of anemia. Increased RDW, indicating the presence of anisocytosis, has been associated to iron deficiency and nutritional deficiencies (folate or vitamin B12) and to a large number of disorders such as cardiovascular disease, venous thromboembolism, cancer, diabetes, community-acquired pneumonia, liver and kidney failure and chronic obstructive pulmonary disease [1]. This comprehensive clinical spectrum makes RDW a parameter with an importance far beyond the differential diagnosis of anemia.

Increased RDW appears as a consequence of deregulation of red blood cells (RBC) homeostasis, involving both impaired erythropoiesis and RBC degradation, and it was recently recognized as a marker of both chronic inflammation and high levels of oxidative stress (OS) [2]. In fact, increased RDW was already related to high levels of pro-inflammatory cytokines, such as tumor necrosis factor α and interleukin 6 [3], high levels of OS-induced RBC damage and shortened RBC survival [4].

Fanconi anemia (FA), the most frequent form of inherited bone marrow failure (BMF), is a recessive/X linked disorder caused by biallelic mutations in one of the 19 FA genes so far characterized [5] that function in a common signaling pathway that controls the maintenance of genomic stability: the *FA/BRCA* pathway. The FA clinical manifestations are heterogeneous, although generally patients progress to BMF which, unless treated, remains the primary cause of morbidity and mortality [6], with patients suffering from early development of cancer, particularly acute myeloid leukemia (AML). At cellular level FA is characterized by chromosome instability (CI) and dysfunctional response to OS. CI is the hallmark of FA, being the hypersensitivity of FA cells to the clastogenic effect of diepoxybutane (DEB) used as a specific diagnostic marker [7]. This hypersensitivity, apart from being an indicator of DNA repair deficiency, may reflect the reduced capacity of FA cells to respond to the OS-related mechanism of DEB cytotoxicity. In fact, there is both in vitro and in vivo evidence indicating that FA cells are in a permanent pro-oxidant state, demonstrated by oxidative DNA damage, increased lipid peroxidation, free iron levels, overproduction of reactive oxygen species (ROS), mitochondrial dysfunction, and glutathione (GSH) depletion [8, 9]. A recent review summarized the latest understanding on the roles of FA proteins in modulating redox functions and the evidence for molecular and clinical involvement of OS in the FA phenotype [10].

Hematological abnormalities occur in virtually all FA patients, but their progression is quite variable. At birth, peripheral blood counts can be normal, but bone marrow may already be hypoplastic and has a reduced pool of CD34+ hematopoietic stem cells (HSCs) [11, 12]. Neonatal aplastic anemia in FA has already been described [13], although hematopoietic dysfunction may not be recognized in infancy because of the significant compensatory mechanisms present in the bone marrow. One of these compensatory mechanisms is stress erythropoiesis, a state of hematopoietic emergency in which the production of erythrocytes is rapidly increased in response to tissue hypoxia, associated to anemia or redox imbalance [14]. Importantly, macrocytosis and increased fetal hemoglobin (HbF), by definition the main indicators of stress erythropoiesis [15, 16], appear among the first parameters that become altered during hematological complications in FA. Anemia is usually the last cytopenia to be expressed.

The significance of RDW and its possible relation to a stress erythropoiesis or a cellular pro-oxidant state have never been explored in FA. The aim of the present study was to analyze the values of RDW in FA and search for a correlation with the hematological parameters most consistently associated to the FA phenotype. The importance of RDW as a marker of stress erythropoiesis is explored.

## Methods

### Subjects

This study does not report on primary research. All clinical data were obtained from protected files resulting from routine diagnosis and treatment, were processed anonymously and are in accordance with the Helsinki Declaration of 1975, as revised in 2000. It included 34 patients (16 males and 18 females) referred for confirmation/exclusion of FA diagnosis based on the DEB test. All patients were diagnosed as FA (DEB+) with a mean age of 10 years (Table 1). 24 patients (P1-P24) presented physical abnormalities compatible with FA and hematologic abnormalities compatible with BMF; 5 patients (P25-P29) presented hematologic abnormalities compatible BMF without physical abnormalities. Five patients (P30-P34) were referred for being FA siblings/relatives.

**Table 1:** Physical, hematological and cytogenetic characterization of 34 Fanconi anemia (FA) patients at diagnosis

FA patients (DEB+)				Presence (+) or absence (−) of hematological abnormalities					Presence (+) or absence (−) of congenital abnormalities					
Patient number	Gender	Age diag	brk/cel	Thromb	Neut	Anem	↑HbF	Macroc	Skin	Growth	Facies	Thumb	Urog	Heart
1	F	8	15.0	+	+	+	+	+	-	+	-	-	-	+
2	M	7	11.3	+	+	+	+	+	+	+	-	-	-	-
3	F	5	1.0	+	+	+	+	+	+	+	-	-	-	-
4	F	4	6.6	+	+	+	+	+	+	-	-	+	-	-
5	F	16	1.5	+	+	+	ND	+	-	+	+	+	-	-
6	M	10	7.0	+	+	+(1)	+	+	+	-	-	+	-	-
7	M	3	7.6	+	-	+(1)	ND	-	-	-	+	+	+	+
8	F	9	6.7	+	+	+(1)	ND	+	-	+	-	+	-	-
9	M	2	13.4	+	+	+	ND	+	-	+	-	+	-	-
10	F	6	7.2	+	+	+	ND	+	-	-	-	+	+	-
11	F	8	4.5	+	+	+	ND	+	-	+	+	-	-	-
12	M	1	3.1	+	-	+	+	+	-	+	+	-	-	-
13	F	3	4.1	+	+	+	ND	+	-	-	+	-	+	-
14	M	8	11.7	+	+	+	+	+	-	+	-	-	-	-
15	F	4	2.2	+	-	+(1)	+	+	-	-	+	-	-	-
16	F	6	2.1	+	+	+	ND	+	+	-	-	-	-	-
17	F	12	1.8	+	+	+	ND	-	+	-	-	-	+	-
18	M	12	1.7	+	+	+(1)	ND	+	+	-	-	-	-	-
19	F	18	9.1	+	+	+	ND	-	-	+	+	-	-	-
20	M	12	1.3	+	+	+	ND	-	-	+	-	-	+	-
21	M	3	3.1	+	-	-	+	-	-	-	-	-	+	+
22	M	25	1.1	-	+	-	ND	+	+	-	-	-	-	-
23	M	1	1.5	-	-	-	+	-	+	-	-	-	-	-
24	F	8	5.4	+	+	+(1)	+	+	+	-	-	-	-	-
25	M	29	9.7	+	+	+	+	+	-	-	-	-	-	-
26	F	21	12.0	+	+	+	+	+	-	-	-	-	-	-
27	F	12	8.6	+	+	+	+	+	-	-	-	-	-	-
28	M	7	4.4	+	+	+	+	-	-	-	-	-	-	-
29	F	17	6.6	+	-	+	ND	+	-	-	-	-	-	-
30*	M	1	7.5	-	+	+	+	-	-	-	+	-	-	-
31*	M	4	7.1	-	+	-	-	-	+	+	-	-	+	-
32*	F	34	7.1	+	+	+	ND	-	-	-	-	-	-	-
33*	F	10	7.1	-	-	-	ND	+	-	-	-	-	-	-
34*	M	0	5.0	-	+	+(2)	ND	-	-	-	-	-	-	-

*FA* Fanconi anemia, *brk/cel* number of DEB (diepoxybutane)-induced breaks per cell; reference values (min-max) for brk/cel in a DEB+ test: 0.96–17.0, *ND* not determined * referred for familial study, *age diag* age, in years, at the time of diagnosis, *thromb* thrombocytopenia, *neut* neutropenia, *anem* anemia, **↑** *HbF* increased fetal hemoglobin, *macroc* macrocytosis, *skin* abnormal skin pigmentation with *café au lait* spots, *growth* small stature, *facies* particular facies, small head and eyes, *thumb* thumb abnormalities, *urog* renal or gonadic abnormalities, *heart* cardiac abnormalities

(1) normal hemoglobin values, decreased red blood cell counts; (2) normal red blood cell counts, decreased hemoglobin

### Cells and cell cultures

From each patient, heparinized peripheral blood was collected at the time of diagnosis, to perform lymphocyte cultures for DEB-induced CI determination, according to a standard protocol [17]. In brief, whole blood (0.5 ml) was cultured in RPMI medium supplemented with 15 % FCS and antibiotics. Cultures were stimulated with phytohemagglutinin (5 μg/ml) and incubated at 37 °C, 5 % CO_2,_ for 72 h. DEB (0.1 μg/ml) was added 24 h after culture initiation. Since DEB is a carcinogen with unknown risk, the appropriate safety precautions were taken [18].

### Cytogenetic analysis

Cells were harvested after 1 h incubation with colcemid (4 μg/ml), followed by hypotonic treatment with 75 mM KCl and fixation in a 1:3 solution of acetic acid:methanol. Chromosome preparations were performed by the standard air drying method, and stained with 4 % Giemsa.

Chromosome aberrations were analyzed by two independent scorers on 50–100 metaphases from coded slides. A minimum of 25 metaphases was counted only when the mitotic index was very low and the rate of CI very high. Each cell was scored for chromosome number and types of structural abnormalities. Tri- tetra-radial figures, dicentric and ring chromosomes were scored as rearrangements and were scored as two breaks. Cells with so much instability that was not possible to count the number of breaks were classified as pulverized cells. CI was determined according to the International Fanconi Anemia Registry (IFAR) protocol [18]; the parameter number of breaks/cell was selected in this study, once it is the discriminating one for the FA diagnosis [7].

### Hematological evaluation

For each patient a complete blood count was determined, for routine purposes, at the time of cytogenetic evaluation. None of them has been subjected to transfusions previously to this evaluation. The following hematological parameters were selected for the present study: red blood cells (RBC) count (×10^6^/μL), hemoglobin (Hb) level (g/dL), neutrophil (Neut) and platelet (Plt) counts (×10^3^/μL), mean corpuscular volume (MCV) value (fL), fetal hemoglobin (HbF) and RDW-CV (coefficient of variation) values (%). Complete blood counts were performed using the system available in each hospital: Advia 2120 (Siemens) or Cell-Dyn (R) Saphire (TM) (Abbott). Those systems don’t measure RDW-SD (standard deviation); they only measure RDW-CV.

Normal ranges (minimum and maximal) were established according to laboratorial reference values, with age and gender adjustments [19]. For 3 patients (P9, P11, P33), with distinct clinical outcomes, a sequential comparative analysis was performed between RDW and Hb values, depicted from routine blood counts determined over 10 years of follow-up (without transfusions or bone marrow transplant).

### Statistical analysis

Statistical analysis was performed using the GraphPad Prism 5. Normality was assessed by Shapiro-Wilk goodness test. Correlation between continuous non-parametric variables was determined by the Spearman test. Comparative analysis between groups was determined using the Mann Whitney test.

## Results

### RDW is increased in most FA patients

**Fig. 1:**
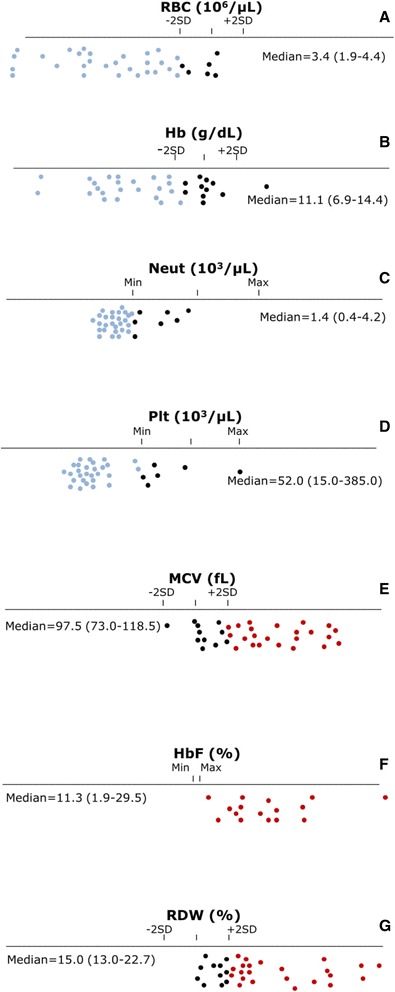
Graphic representation of hematological values from 34 FA patients. Normal ranges with minimum and maximal standard deviation (−2SD, +2SD) or minimal and maximal percentage (Min, Max) were established according to internal laboratorial reference values, with age and gender adjustments. Median value (min-max) of this Fanconi anemia (FA) population is indicated for each parameter. **a** red blood cells (RBC) counts (x10^6^/µL). **b** hemoglobin (Hb) values (%). **c** neutrophil (Neut) counts (x10^3^/µL). **d** platelet (Plt) counts (x10^3^/µL). **e** mean corpuscular volume (MCV) value (fL). **f** fetal hemoglobin (HbF) values (%). **g** red cell distribution width (RDW) values (%)

Hematological parameters commonly used in the characterization of FA were depicted from routine complete blood counts of 34 FA patients, determined at the time of diagnosis. The results showed that 82 % of the patients had decreased RBC values with a median = 3.4 × 10^6^/μL (1.9–4.4) (Fig. 1 a), 68 % had decreased Hb values with a median = 11.1 g/dL (6.9–14.4) (Fig. 1 b), 79 % had decreased Neut values with a median = 1.4 × 10^3^/μL (0.4–4.2) (Fig. 1 c), 82 % had decreased Plt values with a median = 52.0 × 10^3^/μL (15.0–385.0) (Fig. 1 d) and 68 % had increased MCV values with a median = 97.5 fL (73.0–118.5) (Fig. 1 e). HbF was increased in 100 % of the 16 patients so far evaluated, with a median = 11.3 % (1.9–29.5) (Fig. 1 f). RDW values depicted from the same complete blood counts were then evaluated. They were increased in 68 % of the patients, with a median = 15.0 % (13.0–22.7) (Fig. 1 g). RDW increase was independent of gender (*p* = 0.6838) and presence/absence of congenital abnormalities (*p* = 0.2829), and was not correlated with age at diagnosis (*r* = 0.1596, *p* = 0.3672) nor DEB-induced CI (*r* = 0.2795, *p* = 0.1145).

### Increased RDW correlates with anemia, neutropenia and thrombocytopenia in FA patients

**Fig. 2:**
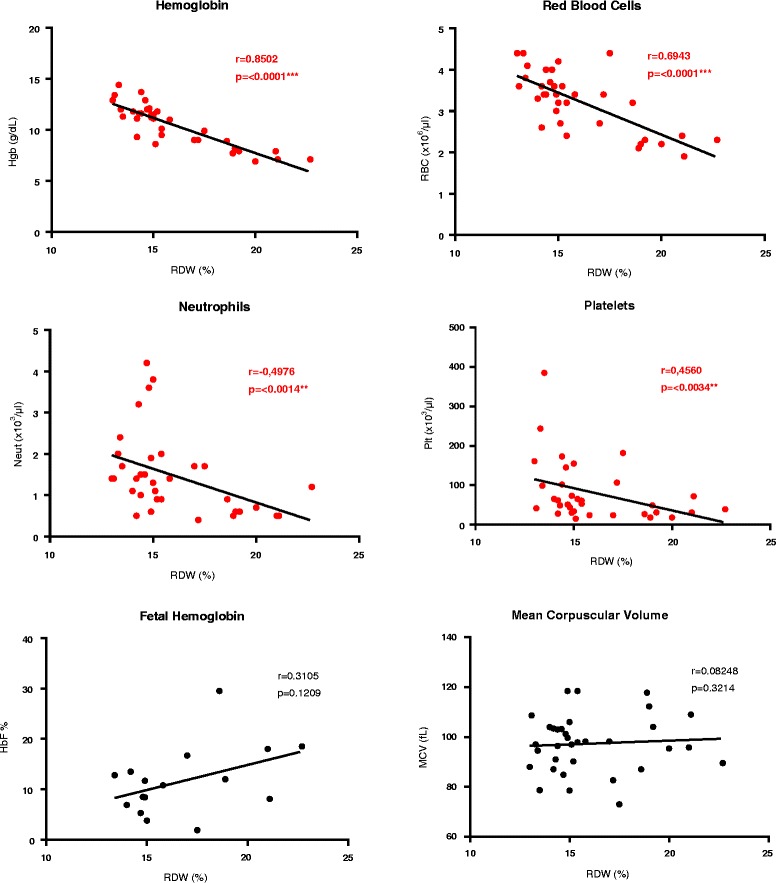
Correlation between RDW hematological parameters most relevant for FA characterization. Red cell distribution width (RDW) values and hematological parameters most relevant for Fanconi anemia (FA) characterization were depicted from routine complete blood counts of 34 FA patients. All values are provided with standard measurement units; *p*-values >0.05 are not significant, *p*-values <0.01 are significant (**) and *p*-values <0.001 are highly significant (***)

In order to better analyze the clinical significance of the increased RDW in FA patients, correlations were performed between RDW and the hematological parameters most relevant for FA characterization. The results showed that RDW from FA patients had a very strong (negative) correlation with Hb (*r* = 0.8502, *p* < 0.0001) (Fig. 2 a), a strong (negative) correlation with RBC (*r* = 0.6943, *p* < 0.0001) (Fig. 2 b) and a moderate (negative) correlation with Neut and Plt (*r* = 0.4976, *p* = 0.0014 and *r* = 0.4560, *p* = 0.0034 respectively) (Fig. 2 c-d). No significant correlations were observed between RDW and HbF or MCV (*p* = 0.1229 and *p* = 0.3214 respectively) (Fig. 2 e-f).

### Correlation between RDW and Hb during follow-up studies of FA patients with different clinical progression

**Fig. 3:**
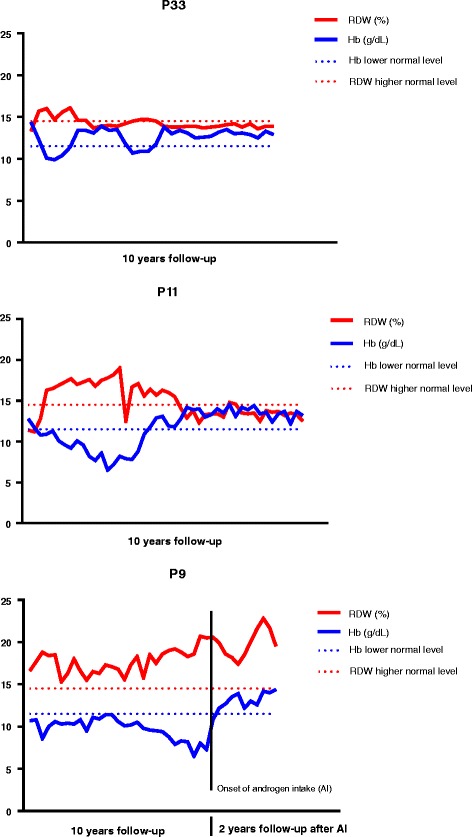
Correlation between RDW and Hb values in sequential follow-up hemograms from 3 FA patients. Red cell distribution width (RDW) values and hemoglobin (Hb) values were depicted from sequential hemograms, routinely evaluated during 10 years follow-up, of 3 Fanconi anemia (FA) patients with different clinical outcomes: an adult patient clinically stable (P32); a young patient with a previous progression to bone marrow failure (BMF) that reverted to a stable condition (P9); a patient with severe BMF since childhood who initiated androgen treatment after 10 years follow-up (P7). All values are provided with standard measurement units

The correlation between RDW and Hb was further explored in sequential hemograms from individual FA patients with different clinical outcomes, not subjected to transfusions or bone marrow transplant: a clinically stable adult patient (P33); a young patient with a previous progression to BMF that reverted to a stable condition (P11); a patient with severe BMF since childhood who initiated androgen treatment (P9) while is waiting for a compatible bone marrow donor. Sequential RDW and Hb variations, routinely evaluated during 10 years follow-up, were compared. Patient P33 (Fig. 3 a), who is clinically stable, showed some anemia events during follow-up, accompanied by proportional increases in RDW values (points 3, 5, 7, 17 and 19 in the x-axis of Fig. 3 a), which then turn to normal values accompanied by normal Hb levels. Patient P11 (Fig. 3 b) showed a severe progression to BMF from 9 to 13 years of age (from points 1 to 16 in the x-axis of Fig. 3 b) and suddenly a spontaneous improvement occurred, with Hb values increasing till normal values accompanied by a decrease in RDW values till a normal range. Patient P9 (Fig. 3 c) presented pancytopenia, with anemia, since 1 year of age, accompanied by increased RDW values. His clinical condition worsened at 10 years when he started an androgen treatment, with oxymetholone (OXM) (at point 32 in the x-axis of Fig. 3 c). The treatment improved significantly the Hb levels; however, RDW values did not decrease to normal values.

## Discussion

### Clinical relevance of increased RDW in FA patients

RDW is a simple parameter that reflects anisocytosis and is traditionally used in the differential diagnosis of anemia. However, increased RDW is also associated to a great number of diseases and, most importantly, associated with overall mortality in the general population [1]. More recently, the prognostic value of increased RDW in coronary artery disease was reviewed, the negative prognostic effects being attributed to the adverse effects of independent risk factors, including OS [20]. Early studies by Nordenson and Joenje established that FA cells present excess oxygen toxicity that leads to OS damage [21, 22]. Since then, several research groups found evidence that FA is characterized by abnormal accumulation of ROS and dysfunctional response to OS [10, 23–33]. Nevertheless, there are no reports in the literature about the importance of RDW in the FA phenotype. In the present study we clearly showed that RDW is significantly increased in most FA patients and is associated with severity of FA disease, i.e., correlated with thrombocytopenia, neutropenia and, most important, highly correlated with anemia.

Hematopoiesis in FA is impaired from the earliest stages of embryonic development [34], and there is a body of evidence supporting the role of dysfunctional HSC biology in the etiology of the disease [35]. Progression to BMF has been considered to be caused by two different mechanisms: the accumulation of OS-induced DNA damage along mitotic divisions, by deficiency in the *FA/BRCA* repair pathway, resulting in p53 activation and apoptosis of the hematopoietic progenitors [36, 37] and overproduction of inhibitory cytokines, which can degrade bone marrow [38], leading to impaired erythropoiesis and RBC degradation. In a recent work a novel connection was established between stress hematopoiesis and the occurrence of DNA damage and functional decline in HSCs: it was shown that DNA damage can be a consequence of the exit of HSCs from their homeostatic quiescent state in response to physiological stress, such as infection [39]. Using a *Fanca*^−^/^−^ mice model, it was shown that repeated activation of HSCs out of their dormant state leads to a complete collapse of the hematopoietic system, which phenocopied the highly penetrant BMF seen in FA patients. All these studies support the hypothesis of a progressive hematopoietic impairment in FA associated with a decreased capacity of the bone marrow to send mature RBC to the periphery, which may lead to RDW increment.

In summary, we showed for the first time that increased RDW is an important clinical marker in FA, with the advantage that this value is easily determined as a part of the standard cell blood count without additional costs. We suggest that increased RDW in FA may be, like in other OS-related disorders, a consequence of deregulation of RBC homeostasis, involving both impaired erythropoiesis and RBC degradation.

### RDW as a marker of stress erythropoiesis

At birth FA patients have normal blood counts, although the bone marrow is hypoplastic and deficient in CD34+ HSCs long before peripheral blood abnormalities appear [11, 12]. Macrocytosis and increased HbF, which are considered markers of stress erythropoiesis [16], are generally the first manifestations to be found in FA peripheral blood cells. Interestingly, in our FA population these two parameters were not correlated with RDW. We postulate that, while macrocytosis and increased HbF reflect a stress erythropoiesis that is responding to bone marrow impairment, increased RDW may reflect a stress erythropoiesis that is responding to a progressive shortened RBC survival at the periphery, which must be compensated by a constant RBC turnover. Therefore, we propose that RDW reflects the need for a recruitment of HSCs to the periphery. This hypothesis is supported by the work of Rodriguez A, Vadillo E, Gonzalez M, Flores P, Sosa D, Torres L, Garcia de Teresa B, Mayani H, Pelayo R and Frias S (unpublished observations, presented at the 26th Annual Fanconi Anemia Research Fund Scientific Symposium, Additional file 1) where it was shown the presence of circulating CD34+ cells and elevated G-CSF levels in the peripheral blood of FA individuals. They suggested that elevated production of G-CSF and the pro-inflammatory bone marrow microenvironment could be responsible for the stress erythropoiesis with mobilization of CD34+ precursor cells to the periphery. According to the present study, we suggest that RDW increment may be the result of a stress erythropoiesis that is responding to an OS-related deregulation of RBC homeostasis and RBC degradation at the periphery, which leads to a mobilization of more immature erythropoietic cells from bone marrow. In accordance, cytoskeleton-dependent alterations were already observed in FA RBC, leading to cell shrinking and blebbing, being hypothesized that these changes may be the result of OS imbalance that leads to alterations in RBC plasticity and deformation-associated functions [40].

### RDW as a marker of hematological disease progression

To better understand if RDW variation can be an important marker during progression of hematological disease, in the present study we evaluated the correlation between RDW and Hb values, obtained from sequential hemograms over 10 years of follow-up, from 3 FA patients with different clinical progressions, not subjected to transfusions or bone marrow transplant. In general, whenever RDW was among normal ranges Hb was also among normal values. RDW increment was systematically correlated with Hb decrease. A graphic model for controlling adverse outcome is proposed, based on the position of sequential RDW and Hb values relatively to reference normal lines (red line for +2SD RDW values and blue line for -2SD Hb values) (Fig. 3). While RDW and Hb values are inside the two reference lines the hematological situation is stable. Whenever RDW and Hb values are outside the two reference lines, the clinical situation worsens. In fact, the adult patient P33, who is now clinically stable, presents RDW and Hb values inside the two reference lines, although during the 10 years follow-up the patient showed some anemia events accompanied by proportional increases in RDW values (values outside the two reference lines). Patient P11 is also stable at present, with RDW and Hb values inside the two reference lines, but after a sudden recovery from a severe situation of BMF, where RDW and Hb values were clearly outside the two reference lines. In fact, at 8 years she presented an unexplained progressive worsening of hematological disease, prolonged for about four years, after which, and in the absence of any treatment, a spontaneous recovery occurred. Sequential evaluation of chromosome instability revealed a reduction in the number of DEB-induced breaks, although not sufficient to be classified as a somatic mosaicism [41], which could explain this new clinical situation (Porto B, personal communication). Patient P9, with a severe condition of BMF since childhood, presented during 10 years follow-up RDW and Hb values always outside the two reference lines, with a progressive detachment between the two values. When the patient started OXM treatment, Hb immediately increased up to normal values. However, Hb increase was not accompanied by RDW decrease. We hypothesize that erythropoiesis, for some reason, continues to be stressed. Androgens are widely used for FA treatment, but their mode of action is not completely understood. In a recent study [42] aged *Fancd2*^−^/^−^ mice were used to assess the therapeutic efficacy of OXM. Chronic OXM treatment significantly improved hematological parameters, decreasing macrocytosis and pancytopenia, and stimulated the proliferation of hematopoietic stem and progenitor cells. However, competitive repopulation assays demonstrated that this therapy eventually results in stem cell exhaustion, which may have direct clinical implications for the treatment of BMF. In agreement with these results, we hypothesize that, in the case of patient P9, the fact that Hb demand is not accompanied by RDW decrease may probably lead to a risk of HSCs exhaustion in the long term.

Our study has two main limitations: the RDW value, per se, is not a diagnostic marker, because some patients, with a mild hematological disease, can still have normal RDW values. Therefore, its main importance is related to the progression of hematological disease, as shown by the present results. Additionally, as the follow-up data only relates to three patients, the hypothesis regarding the use of RDW to predict severity of disease and adverse outcomes must be evaluated in a future work, with a higher number of patients included.

## Conclusion

In conclusion, the present study provides an important and novel clinical finding that may have importance for the follow-up of FA patients: it was shown, for the first time, that RDW is increased in FA patients and this increment is related with progression of hematological disease, in particular progression to anemia. It is suggested that increased RDW can be a novel marker of stress erythropoiesis in FA.

## Abbreviations

AML, acute myeloid leukemia; BMF, bone marrow failure; CI, chromosome instability; DEB, diepoxybutane; FA, Fanconi anemia; GSH, glutathione; Hb, hemoglobin; HbF, fetal hemoglobin; HSC, Hematopoietic stem cell; IFAR, International Fanconi Anemia Registry; MCV, mean corpuscular volume; Neut, neutrophils; OS, oxidative stress; OXM, oxymetholone; Plt, platelets; RBC, red blood cells; RDW, red cell distribution width; ROS, reactive oxygem species; SE, stress erythropoiesis

## Additional file

Additional file 1: Required informations about the unpublished data referred in the main manuscript. (PDF 463 kb)
