# Supplementary material for: Increased red cell distribution width in Fanconi anemia: a novel marker of stress erythropoiesis
Source: Orphanet J Rare Dis. 2016 Jul 25;11:102. doi: 10.1186/s13023-016-0485-0 (PMC4960735; doi:10.1186/s13023-016-0485-0)
Supplement: Additional file 1: — Required informations about the unpublished data referred in the main manuscript. (PDF 463 kb) [file 13023_2016_485_MOESM1_ESM.pdf]

Supplemental File

Required informations about the unpublished data referred in the main manuscript.

11:45 - 11:50 **Session Wrap-up: Richard Gelinas, PhD**

11:50 - 1:50

Lunch Buffet ***(served at 11:50)***
Grand Ballroom Salons F-H
Joint Board of Directors and Scientific Advisory Board Meeting
Brookside (Lower Level)
Poster Viewing
Grand Ballroom Salon E

Session VII: Expanding FA Clinical Phenotype

Chair: Akiko Shimamura, MD, PhD
Fred Hutchinson Cancer Research Center, Seattle, Washington

1:50 - 1:55 **Session Overview: Akiko Shimamura, MD, PhD**

1:55 - 2:05 **Jason Taylor, MD, PhD**, Oregon Health & Science University, Portland, Oregon: *Fancc-/- Mice have Decreased Bone Mineral Density (BMD) that is the Result of Decreased Trabecular Bone*

2:05 - 2:10 Questions and Answers

2:10 - 2:20 **Emmy Verheij, MD**, University Medical Center Utrecht, Utrecht, Netherlands: *Prevalence of Hearing Loss and Speech in Noise Difficulties in Fanconi Anemia*

2:20 - 2:25 Questions and Answers

2:25 - 2:35 **Alfredo Rodríguez Gómez, MSc,** Instituto Nacional de Pediatría, Mexico City, Mexico: *Presence of Circulating CD34+ Cells with Multi-lineage Differentiation Potential in the Peripheral Blood of FA Individuals*

2:35 - 2:40 Questions and Answers

2:40 - 2:45 **Session Wrap-up: Akiko Shimamura, MD, PhD**

Session VIII: Mediators of FA Pathway Function

Chair: Agata Smogorzewska, MD, PhD
The Rockefeller University, New York, New York

2:45 - 2:50 **Session Overview: Agata Smogorzewska, MD, PhD**

2:50 - 3:00 **Audrey Magron, MSc, PhD Student**, Laval University, Québec, Canada: *The Regulation of STMN-1 Phosphorylation in the Context of Fanconi Anemia: Consequence on Cellular Division*

3:00 - 3:05 Questions and Answers

Presence of Circulating CD34+ Cells with Multi-lineage Differentiation Potential in the Peripheral Blood of FA Individuals

A. Rodriguez[1], E. Vadillo[2], M. Gonzalez[1], P. Flores[3], D. Sosa[1], L. Torres[1], B. Garcia de Teresa[1], H. Mayani[3], R. Pelayo[3], S. Frias[1,2]

[1]Instituto Nacional de Pediatria, Mexico D.F., Mexico; [2]Instituto de Investigaciones Biomedicas, UNAM, Mexico D.F., Mexico; [3]UIMEO, Centro Medico Nacional Siglo XXI, Mexico D.F,. Mexico

Introduction: Fanconi anemia (FA) is a chromosome instability syndrome with congenital malformations, cancer predisposition and progressive bone marrow failure (BMF). BMF in FA patients is considered to be due to massive apoptosis of the CD34+ hematopoietic compartment, however, additional factors could contribute to BMF such as the detachment of CD34+ cells from its hematopoietic niche caused by inflammation. In this study, we detected circulating CD34+ present in peripheral blood of FA patients and characterized them.

Methods: Peripheral blood from FA patients was obtained. CD34+ cells were detected in the peripheral mononuclear cell fraction by FACS, they were isolated by positive selection and set in different culture conditions to detect their proliferation and differentiation potential towards myeloid, erythroid and lymphoid lineages. Cytokines production and apoptotic status were also evaluated.

Results: The peripheral blood of 18 FA patients and 12 age-paired healthy individuals were studied. CD34+ cells were found to be enriched up to ten times in FA patients. Remarkably, the younger FA individuals had a higher number of circulating CD34+ cells, whilst older FA individuals (usually pancytopenic) have less. FA circulating CD34+ cells showed a limited in vitro proliferation potential, but they preserved their differentiation potential towards myeloid, erythroid, NK and T-lymphoid lineages, demonstrating their progenitor identity. However B-lymphoid lineage had the more affected proliferation potential. Although CD34+ culture supernatants appeared heterogeneous with respect to TNFα, IL-6 and IL-1β content, G-CSF levels, a well-known CD34+ mobilizing cytokine, appeared constantly elevated.

Conclusion: Elevated production of G-CSF and the pro-inflammatory BM microenvironment could lead to mobilization of CD34+ precursor cells with lymphoid and myeloid differentiation potentials in FA individuals. These cells showed to have an impaired proliferation and unequal differentiation potential probably due to DNA damage accumulation and pro-apoptotic status. The detaching of CD34+ cells from their hematopoietic niche might be an additional factor contributing to BMF in FA individuals..

Translational Applicability: These findings have implications in the recognition of factors leading to pancytopenia, as well as the potential of these readily available circulating CD34+ cells to be used for cell and gene therapy.
